# “B” Noticed

**Published:** 1858-06

**Authors:** A. Berry


### "B" NOTICED.

The truth of the saying, that there is but one step from the sublime to the ridiculous, is strikingly exemplified in our friend, Dr. Brown's article on "Cheoplasty and its Results," and "Novice Noticed."

With the temerity of a fledgling he is proudly soaring in the pure empyrean around the pinnacles of fame, hymning the praises of the inventor of Cheoplasty, and hoping that soon the "whole profession will join in swelling the notes of praise to the author of this invaluable compound," when a squib from "Novice," brings him down floundering in the murky atmosphere, among jackasses. What a fall! Poor fellow, hope his wings are not broken.

"A reckless scribbler, skulking under cover of an anonymous signature, with the very perfection of impudence, and with unmistakable indications of gross ignorance," "gnarling and sneering," "dull stupidity." Does not our friend know that the use of this language, under the circumstances, is quite improper for a "gentleman?" Is he not aware that if he had studied his favorite assology less, and the sciences pertaining to his profession more, he might not have given us the bombastic nonsense and humbuggery, that called forth the good-natured and appropriate criticism of "Novice." Why instead of this talk to Buncombe, did not our friend hold his "asses," and favor us further about the *purity* of the cheoplastic compound? Why not enlighten us more fully as to a mixture of tin, &c., "possessing the same non-corrosive property as gold?" Why not furnish specifications respecting Cheoplasty having "no spaces left, in which food and secretions can lodge, hence, for comfort, cleanliness and utility it stands unrivalled;" while in fact, as to *no spaces left*, it is identical with the continuous gum, and the tin work of former years.

Notwithstanding his pointed remarks on the importance of "real names," he appends *only his initials*, to his "Novice Noticed." Was Dr. Brown ashamed of his bantling, so that he disliked to give his name in connection with it?

Novice is not disposed to undervalue the efforts of the clergy, in behalf of science; but nostrum puffing is not always promoting science.

Our friend informs us that he has received a fine book from a friend. Novice would respectfully suggest to him that a careful study of its style might improve his own.

Should an ignoramus, inflated with self-conceit, set up as promulgator of dental science, and make such ridiculous assertions as our friends article on "Cheoplastry and its Results" contained, he ought to bear a stricture like Novice's with a show of equanimity. Especially was it proper for our astute and learned friend, with his advantages of association with distinguished ministers, to have kept cool.

The efforts to doubt the truth of my statement, of the now use of Cheoplasty in St. Louis and Cincinnati—letters "just such as the occasion required," would have been interesting.

With his usual regard for varacity, he gives us "clever ones" to be understood as used by me—the "*ones*" was not in my article.

His lamentation of the direful condition of the profession in the West, if they take the *ipse dixit* of Novice, and are held back from the use of Cheoplasty by prejudice, selfishness and bigotry, are wholly gratuitous. If, after being dosed *ad nauseam*, for a twelve-month, with certificates with "real names," they are not convinced, surely the *ipse dixit* of Novice will not greatly set them back. The profession in the West are much given to doing their own thinking, and generally examine for themselves the wares offered in the market, no matter whether they are wooden-nutmegs, or patented tin, &c., for dental purposes.

Our friend's "clever ones," who abandoned the use of Cheoplasty, will compare not unfavorably with his "Gentlemen" at Baltimore and elsewhere, who are so fond of it. In connection with this assumption of Eastern superiority, it may not be out of place to remark, that if the discussions of the American Dental Convention are a criterion of acquirement, the profession in the West are at least a dozen years in advance of their Eastern brethern. Salt-water atmosphere seems to make other things green besides trunk locks. To be sure the members of our profession down East are gentlemen all, but some of them draw too largely on their imaginations, in describing the wonders of Cheoplasty.

Our friend will permit Novice to assure him that he hopes that his future flights will not extend so far into the clouds—that he will not see so far through " gloomy mists," or at least that he will give us a more subdued account of what he sees. A. Berry, D. D. S.

March 30, 1858.

---

